# A novel quantitative real-time polymerase chain reaction method for detecting toxigenic *Pasteurella multocida* in nasal swabs from swine

**DOI:** 10.1186/s13028-016-0267-7

**Published:** 2016-12-01

**Authors:** Simone Scherrer, Daniel Frei, Max Michael Wittenbrink

**Affiliations:** Vetsuisse Faculty, Institute of Veterinary Bacteriology, University of Zurich, Winterthurerstrasse 270, 8057 Zurich, Switzerland

**Keywords:** Pig, Progressive atrophic rhinitis, *Pasteurella multocida*, *toxA* gene, Quantitative real-time PCR

## Abstract

**Background:**

Progressive atrophic rhinitis (PAR) in pigs is caused by toxigenic *Pasteurella multocida*. In Switzerland, PAR is monitored by selective culture of nasal swabs and subsequent polymerase chain reaction (PCR) screening of bacterial colonies for the *P.* *multocida toxA* gene. A panel of 203 nasal swabs from a recent PAR outbreak were used to evaluate a novel quantitative real-time PCR for toxigenic *P.* *multocida* in porcine nasal swabs.

**Results:**

In comparison to the conventional PCR with a limit of detection of 100 genome equivalents per PCR reaction, the real-time PCR had a limit of detection of 10 genome equivalents. The real-time PCR detected *toxA*-positive *P.* *multocida* in 101 samples (49.8%), whereas the conventional PCR was less sensitive with 90 *toxA*-positive samples (44.3%). In comparison to the real-time PCR, 5.4% of the *toxA*-positive samples revealed unevaluable results by conventional PCR.

**Conclusions:**

The approach of culture-coupled *toxA* PCR for the monitoring of PAR in pigs is substantially improved by a novel quantitative real-time PCR.

**Electronic supplementary material:**

The online version of this article (doi:10.1186/s13028-016-0267-7) contains supplementary material, which is available to authorized users.

## Findings

Progressive atrophic rhinitis (PAR) in pigs is caused by toxigenic *Pasteurella multocida* (capsule types A and D) which synthesize a 145-kDa toxin (dermonecrotic toxin) [1] encoded by the chromosomal *toxA* gene [2, 3]. PAR is of worldwide economic importance to swine production due to a reduction in growth rate in fattening pigs. In Switzerland, PAR is monitored in about 90% of the Swiss primary nucleus and secondary multiplying herds by annual bacteriological examination of 10 nasal swabs per herd for toxigenic *P.* *multocida*. Swabs are analysed by culturing the swabs on selective agar [4] and subsequent screening of bacterial colonies for toxigenic *P.* *multocida* by polymerase chain reaction (PCR) [5]. During a recent outbreak of PAR in Swiss multiplying herds [6], nasal swabs from feeder pigs were tested for toxigenic *P.* *multocida* by the method outlined above. A panel of 203 nasal swabs from this PAR outbreak were further used to evaluate a novel quantitative real-time PCR (qRT-PCR) for toxigenic *P.* *multocida* in porcine nasal swabs. The bacterial strains used in this study are given in Table 1. Strains were grown on 7% sheep blood agar aerobically at 37 °C for 24 h (PB5008A, Oxoid, Pratteln, Switzerland).

**Table 1:** Reference strains used in this study

Organism	Strain	Source	*toxA* gene
*Pasteurella multocida* ssp. *multocida*	ATCC 12948	Pig	+
*Pasteurella multocida* ssp. *multocida*	ZH 1760	Pig nose	+
*Pasteurella multocida* ssp. *multocida*	ZH 17056-18	Pig nose	+
*Pasteurella multocida*	ZH 2232	Pig nose	+
*Pasteurella multocida*	ZH 148	Sheep lung	+
*Pasteurella canis*	ZH 401	Cat wound	+
*Pasteurella multocida* ssp. *multocida*	ATCC 43137	Pig	–
*Pasteurella canis*	ATCC 43326	Dog throat	–
*Pasteurella multocida*	ZH 1950	Pig nose	–
*Pasteurella multocida*	ZH 1949	Pig nose	–
*Pasteurella multocida*	ZH 17430	Cow lung	–
*Pasteurella multocida*	ZH 1615	Cow nose	–

Nasal swabs were collected from a total of 203 feeder pigs on farms affected by PAR [6]. Cotton swabs were transported in Amies medium in screw cap plastic vials (VWR, Dietikon, Switzerland) to the lab. Swabs were streaked onto selective blood agar plates according to Rutter et al. [4] (PB5175A, Oxoid, Switzerland), incubated for 24 h at 37 °C under 5% CO_2_. From each agar plate *Pasteurella*-like colonies or if necessary an arbitrarily taken fraction from the bacterial lawn were subjected to DNA extraction.

Genomic DNA was released by heat lysis of bacterial colonies resuspended in water on a thermal shaker for 10 min at 99 °C. After centrifugation at 17,000*g* for 3 min the supernatant was transferred into a fresh tube and stored at –20 °C until further use. Bacteria from nasal swab cultures were processed accordingly.

Primer and probes were selected using Primer Express Software v3.0.1 (Applied Biosystems, Zug, Switzerland) from alignments of the available sequences of the *P.* *multocida toxA* gene (GenBank accession numbers: AF240778.1, AY864768.1, EF441531.1, X51512.1, X52478.1 and FN398148.1). According to pairwise comparison of the *toxA* gene the percent identity were between 99.76 and 100% underlining its high degree of conservation. The primers selected amplify a conserved 86 base pair (bp) fragment within the 3858 bp *toxA* gene (GenBank accession number: AF240778.1) between nucleotides 2097 and 2182 (Table 2).

**Table 2:** Primer and probe sequences with their respective dye and quencher used for the qRT-PCR

Primer/probe	Target	Concentration	Sequence 5′-->3′
*toxA*			
toxA-F	toxA	0.4 µM	GAAATGGCTGGAAAAACCAGTG
toxA-R	toxA	0.4 µM	GAAAAGGCGCTGAAATTACTGTATC
toxA-probe	toxA	200 nM	**5-FAM-**CGGCTGATTTAATACGCTTTGCCTTGC**–BHQ-1**
Internal control			
eGFP-1-F	eGFP	0.2 µM	GACCACTACCAGCAGAACAC
eGFP-10-R	eGFP	0.2 µM	GAACTCCAGCAGGACCATG
eGFP-probe	eGFP	25 nM	**ATTO 647** **N**-AGCACCCAGTCCGCCCTGAGCA-**BHQ-3**

Gene specificity of both primers and the probe were confirmed by BLAST searches. Primers were synthesized by Microsynth (Balgach, Switzerland). DNA probes were supplied by Eurogentec S.A. (Seraing, Belgium). The probes were quenched by black-hole non-fluorescent quenchers at the 3′-end. Rox dye (Life Technologies, Darmstadt, Germany) was used as an internal reference for normalization and data analyses. An internal amplification control (IAC) was introduced for monitoring each reaction, since bacterial lysates could contain inhibitory substances. Five femtogram (fg) of the pEGFP-1 strandard vector (BD Bioscience Clonetech, USA) was used as IAC template, and a 177 bp amplicon [7] was amplified with primers and the probe given in Table 2. DNA (1 pg) from a *toxA*-positive and a-negative *P.* *multocida* reference strain (ATCC 12948, ATCC 43137) were used as controls in each qRT-PCR run.

The qRT-PCR was performed on an ABI 7500 Fast Real-Time PCR Instrument (Applied Biosystems) using the Path-ID™ qPCR Master Mix (2×) (Life Technologies). Each reaction contained 5 µl master mix, 200 nM of each eGFP-1-F and eGFP-10-R primer [7], 1 µl IAC eGFP plasmid DNA (5 fg), 25 nM ATTO 647 N-labeled eGFP probe, 400 nM of each toxA-F and toxA-R primer, 200 nM FAM labeled toxA probe, 2 µl template DNA and nuclease free water to a final reaction volume of 10 µl.

Cycling conditions were 10 min at 95 °C, followed by a two-step cycling stage of 40 cycles of 15 s at 95 °C and 60 s at 60 °C. Data analysis was handled by the 7500 Software version 2.0.4 (Life Technologies). Samples were considered positive when presenting a typical amplification curve with a Ct value of ≤38 for *toxA* and a Ct value of ≤32 for the IAC. Analyses of samples with IAC Ct values >32 were repeated after reduction of PCR-inhibitory substances by 1:2 and 1:10 dilution.

With an estimated genome size of 2.26 Mb a DNA quantity of 2.5 fg was calculated for one genome equivalent (GE) of *P.* *multocida* [8]. The efficiency of the qRT-PCR was determined by plotting standard curves (Ct values against quantified GE) in 10-fold dilution from 2 × 10^7^ to 2 × 10^3^ GE. The slope of the linear relationship of this curve was used to calculate the amplification efficiency [9]. To determine the minimum detectable bacterial concentrations for the qRT-PCR, twelve replicates of a DNA dilution series containing 4000, 400, 40, 20, 10, 5 and 2 GE of *P.* *multocida* ATCC 12948 per 10 μl PCR reaction were analysed. As illustrated in Table 3, the limit of detection (LOD) was determined to be 10 GE, the lowest dilution for which the acceptance criteria (Ct <38 and a standard deviation of <0.5) were fulfilled.

**Table 3:** Determination of the LOD of the qRT-PCR

Target	Genome equivalents *P. multocida* ATCC 12948	Ct	SD
A. *toxA* (FAM)	2	37.14	0.77
	5	36.06	0.58
	*10*	*34.74*	*0.37*
	20	33.80	0.37
	40	32.78	0.21
	400	29.30	0.17
	4000	25.91	0.23
B. *eGFP* (ATTO 647N)	2	28.12	0.15
	5	28.04	0.16
	10	27.98	0.21
	20	28.05	0.19
	40	28.03	0.27
	400	27.99	0.18
	4000	27.94	0.14

Ct values using DNA template of the *P. multocida* ATCC 12948 reference strain and its standard deviation (SD) of a series of 12 replicates were determined for each dilution step ranging from 2 to 4,000 genome equivalents. *A* Ct values of the *toxA* measured by the FAM detection channel are represented whereas in (*B*) Ct values of the internal amplification control measured by the Cy5 detection channel are shown

The sensitivity of both the qRT-PCR and the conventional PCR assays was confirmed by testing *toxA*-positive *P.* *multocida* strains. The specificity of the qRT-PCR was confirmed by analysis of 32 different bacterial species (Additional file 1).

An amplification plot of a dilution series of toxA was created (Additional file 2). The efficiency of the qRT-PCR was determined by the use of serial dilution standard curves. The linear correlation coefficient r^2^ for the *toxA* gene target was 0.999, showing a high degree of linearity of the qRT-PCR. Based on the slope of the standard curve (–3.348), the amplification efficiency was calculated as 98.9%. At DNA template amounts >1μg, the eGFP amplification was reduced and was completely inhibited with 1.8 μg template DNA. For diagnostic application of the qRT-PCR, a DNA template of maximum 1 μg DNA template was used. To determine the LOD of the assay the acceptance criteria of Ct < 38 and a standard deviation of <0.5 were used. With a LOD of 10 GE, the qRT-PCR showed a tenfold higher sensitivity than the conventional PCR with a LOD of on average 100 GE per PCR reaction [5]. The specificity of both PCR assays was confirmed by repeated test of *toxA*-positive *P.* *multocida* strains. The panel of 32 heterologous bacteria scored negative (Additional file 1). Overall, the qRT-PCR assay revealed a high precision as confirmed by the inter- and intra-assay coefficient of variations (CV) of the Ct values of the positive control as well as the IAC from 52 independent PCR runs. The CV inter-assay values were 5.9% for toxA and 2.7% for IAC; the intra-assay CV values were 1.1% for toxA and 0.58% for IAC.

By comparative analysis of 203 nasal swabs from pigs suspected for PAR, 90 samples (44.3%) were identified as *toxA* gene-positive by both PCR protocols. A sample of 11 swabs (5.4%) were identified as *toxA* gene-positive by qRT-PCR (Ct values 16–33.5), whereas the conventional PCR revealed unevaluable results (e.g. several PCR amplifications products of different but unexpected sizes) despite the positive and negative control reactions performed as expected. Overall, the qRT-PCR detected *toxA*-positive *P.* *multocida* in 101 swabs (49.8%). By comparison, the conventional PCR was less sensitive with 90 samples (44.3%) identified as *toxA*-positive *P.* *multocida*.

Noteworthily, a *Pasteurella canis* strain from the skin wound of a cat was identified as *toxA*-positive by our novel qRT-PCR. This finding was verified by two independent PCR reactions: Species identification was confirmed by the *P.* *canis*-specific PCR of Krol et al. [10], targeting *sodA*, a housekeeping gene encoding manganese-dependent superoxide dismutase. In addition to the PCR species identification, strain ZH 401 was confirmed as *P. canis* by MALDI-TOF analysis. Moreover, a partial fragment of *toxA* from *P. canis* ZH 401 has been sequenced and shows a few point mutations in comparison to the highly conserved sequences of *toxA*-positive *P. multocida* strains (Additional file 3). The presence of *toxA* in our feline *P.* *canis* was confirmed with an in-house *toxA*-PCR (primer 34_ToxA 5′ ACTGTAAAAGGAAAAAGTGCCGATG 3′ and 35_ToxA_rev 5′ AAGAGGAGGCATGAAGAGTGC 3′) resulting in a 3792 bp *toxA* specific amplicon. The expression of toxA in strain ZH 401 was confirmed with the aid of a *P.* *multocida* toxin (PMT) ELISA (Oxoid, Pratteln, Switzerland). The finding of *toxA* in other bacteria than *P. multocida* is of particular interest. Since *toxA* is encoded within a lysogenic prophage, it may be transferred to other bacteria (as in our case to *P.* *canis*) by transduction [11].

To conclude, our novel qRT-PCR is highly efficient and robust to diagnose *toxA*-positive *P.* *multocida* in nasal swabs. Compared with the conventional PCR, the qRT-PCR is more sensitive and specific and time saving. This qRT-PCR will facilitate large scale screening to monitor PAR in swine populations.

## Additional files

**Additional file 1.** A panel of 32 bacteria strains used for specificity testing of the qRT-PCR.

**Additional file 2.** qRT-PCR amplification plot for toxigenic *Pasteurella multocida.*

**Additional file 3.** Alignment of *toxA*-positive *Pasteurella* spp. Swiss field strains.

## References

[1] Chanter N, Rutter JM, Mackenzie A (1986). Partial purification of an osteolytic toxin from *Pasteurella multocida*. J Gen Microbiol.

[2] Buys WE, Smith HE, Kamps AM, Kamp EM, Smits MA (1990). Sequence of the dermonecrotic toxin of *Pasteurella multocida* ssp*. multocida*. Nucleic Acids Res.

[3] Petersen SK (1990). The complete nucleotide sequence of the *Pasteurella multocida* toxin gene and evidence for a transcriptional repressor, TxaR. Mol Microbiol..

[4] Rutter JM, Taylor RJ, Crighton WG, Robertson IB, Benson JA (1984). Epidemiological study of *Pasteurella multocida* and *Bordetella bronchiseptica* in atrophic rhinitis. Vet Rec..

[5] Lichtensteiger CA, Steenbergen SM, Lee RM, Polson DD, Vimr ER (1996). Direct PCR analysis for toxigenic *Pasteurella multocida*. J Clin Microbiol.

[6] Nathues C, Estermann A, Prohaska S, Huber H, Zimmermann W, Schüpbach-Regula G. Outbreak investigation of progressive atrophic rhinitis in an SPF pig population. Vet Epidemiol Econ. 2012.

[7] Hoffmann B, Depner K, Schirrmeier H, Beer M (2006). A universal heterologous internal control system for duplex real-time RT-PCR assays used in a detection system for pestiviruses. J Virol Methods.

[8] May BJ, Zhang Q, Li LL, Paustian ML, Whittam TS, Kapur V (2001). Complete genomic sequence of *Pasteurella multocida*, Pm70. Proc Natl Acad Sci USA..

[9] Klein D, Janda P, Steinborn R, Muller M, Salmons B, Gunzburg WH (1999). Proviral load determination of different feline immunodeficiency virus isolates using real-time polymerase chain reaction: influence of mismatches on quantification. Electrophoresis.

[10] Krol J, Bania J, Florek M, Pliszczak-Krol A, Staroniewicz Z (2011). Polymerase chain reaction-based identification of clinically relevant *Pasteurellaceae* isolated from cats and dogs in Poland. J Vet Diagn Invest.

[11] Pullinger GD, Bevir T, Lax AJ (2004). The *Pasteurella multocida* toxin is encoded within a lysogenic bacteriophage. Mol Microbiol.

