# Supplementary material for: A novel quantitative real-time polymerase chain reaction method for detecting toxigenic Pasteurella multocida in nasal swabs from swine
Source: Acta Vet Scand. 2016 Dec 1;58:83. doi: 10.1186/s13028-016-0267-7 (PMC5131409; doi:10.1186/s13028-016-0267-7)
Supplement: Supplementary file 1 — Additional file 1. A panel of 32 bacteria strains used for specificity testing of the qRT-PCR. [file 13028_2016_267_MOESM1_ESM.docx]

**Additional file 1** A panel of 32 bacteria strains used for specificity testing of the qRT-PCR

| **Organism** | **Source/Strain** |
| --- | --- |
| *Actinobacillus pleuropneumoniae* | ATCC 27088 |
| *Haemophilus parasuis* | ATCC 19417 |
| *Pasteurella mairii* | Clinical isolate |
| *Klebsiella pneumoniae* | Clinical isolate |
| *Trueperella pyogenes* | DSM 20630 |
| *Yersinia enterocolitica* | DSM 11502 |
| *Streptococcus equi spp. Equi* | Clinical isolate |
| *Aeromonas hydrophila* | DSM 6173 |
| *Campylobacter coli* | DSM 4689 |
| *Campylobacter jejuni* | DSM 4688 |
| *Clostridium perfringens Typ A* | DSM 756 |
| *Pseudomonas aeruginosa* | ATCC 27853 |
| *Staphylococcus aureus* | ATCC 25923 |
| *Enterococcus faecalis* | ATCC 29212 |
| *Escherichia coli* | Clinical isolate |
| *Staphylococcus aureus, MRSA* | ATCC 43300 |
| *Enteroccoccus faecalis, VRE* | ATCC 51299 |
| *Corynebacterium pseudotuberculosis* | ATCC 19410 |
| *Staphylococcus intermedius* | DSM 20373 |
| *Staphylococcus pseudintermedius* | DSM 21284 |
| *Rhodococcus equi* | DSM 20307 |
| *Streptococcus agalactiae* | DSM 2134 |
| *Corynebacterium renale* | DSM 20688 |
| *Bordetella bronchiseptica* | Clinical isolate |
| *Streptococcus suis* | Clinical isolate |
| *Moraxella bovis* | Clinical isolate |
| *Staphylococcus chromogenes* | Clinical isolate |
| *Erysipelothrix rhusiopathiae* | Clinical isolate |
| *Staphylococcus hyicus* | Clinical isolate |
| *Mannheimia haemolytica* | Clinical isolate |
| *Streptococcus canis* | Clinical isolate |
| *Acinetobacter baumanii* | Clinical isolate |

ATCC, American Type Culture Collection; DSM, Deutsche Sammlung von Mikroorganismen (German Collection of Microorganisms)
