# Supplementary material for: A novel quantitative real-time polymerase chain reaction method for detecting toxigenic Pasteurella multocida in nasal swabs from swine
Source: Acta Vet Scand. 2016 Dec 1;58:83. doi: 10.1186/s13028-016-0267-7 (PMC5131409; doi:10.1186/s13028-016-0267-7)
Supplement: Supplementary file 2 — Additional file 2. qRT-PCR amplification plot for toxigenic Pasteurella multocida. [file 13028_2016_267_MOESM2_ESM.pptx]

## Slide 1
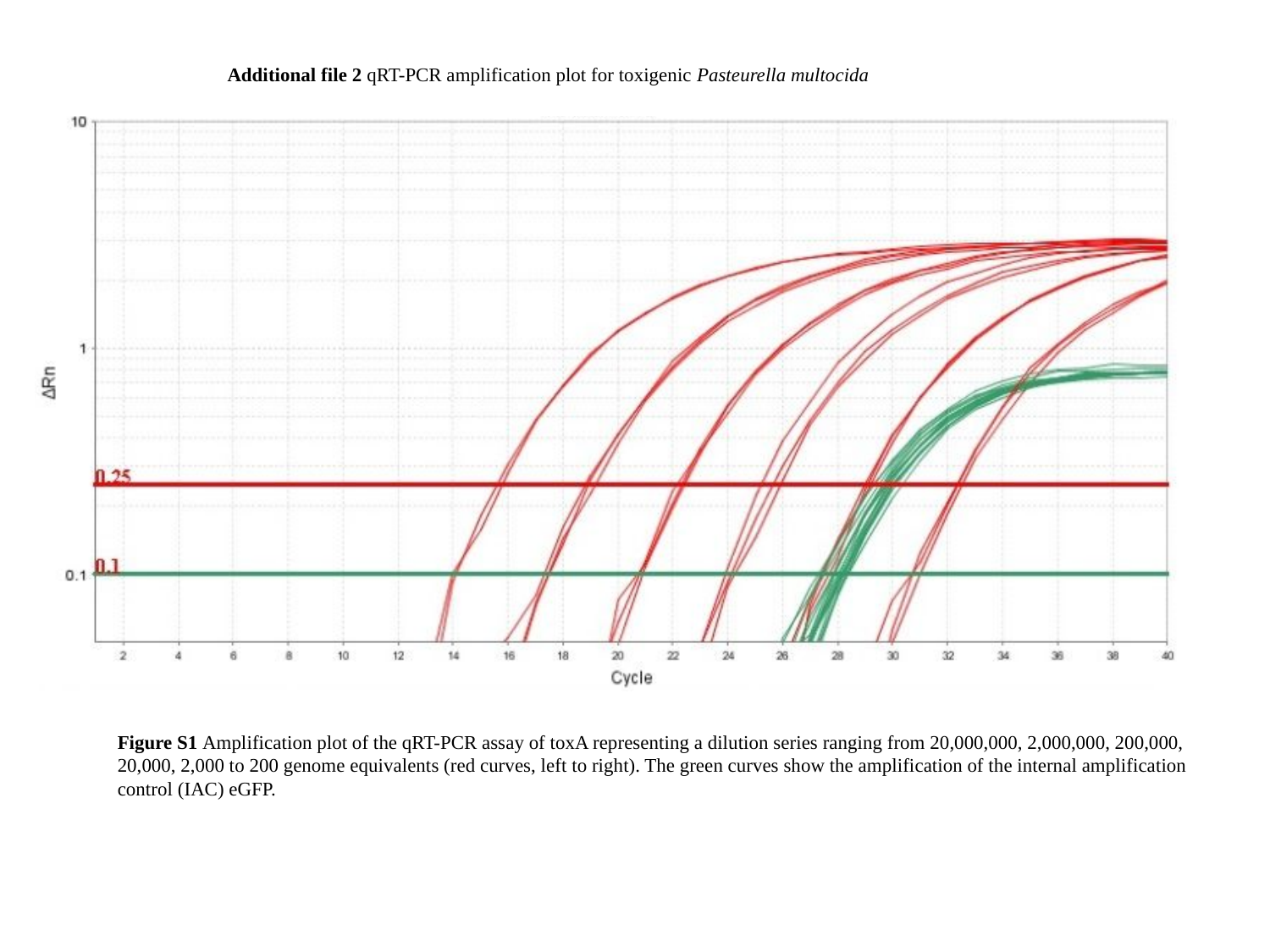

Additional file 2 qRT-PCR amplification plot for toxigenic Pasteurella multocida
Figure S1 Amplification plot of the qRT-PCR assay of toxA representing a dilution series ranging from 20,000,000, 2,000,000, 200,000, 20,000, 2,000 to 200 genome equivalents (red curves, left to right). The green curves show the amplification of the internal amplification control (IAC) eGFP.
