# Supplementary material for: A novel quantitative real-time polymerase chain reaction method for detecting toxigenic Pasteurella multocida in nasal swabs from swine
Source: Acta Vet Scand. 2016 Dec 1;58:83. doi: 10.1186/s13028-016-0267-7 (PMC5131409; doi:10.1186/s13028-016-0267-7)
Supplement: Supplementary file 3 — Additional file 3. Alignment of toxA-positive Pasteurella spp. Swiss field strains. [file 13028_2016_267_MOESM3_ESM.pptx]

## Slide 1
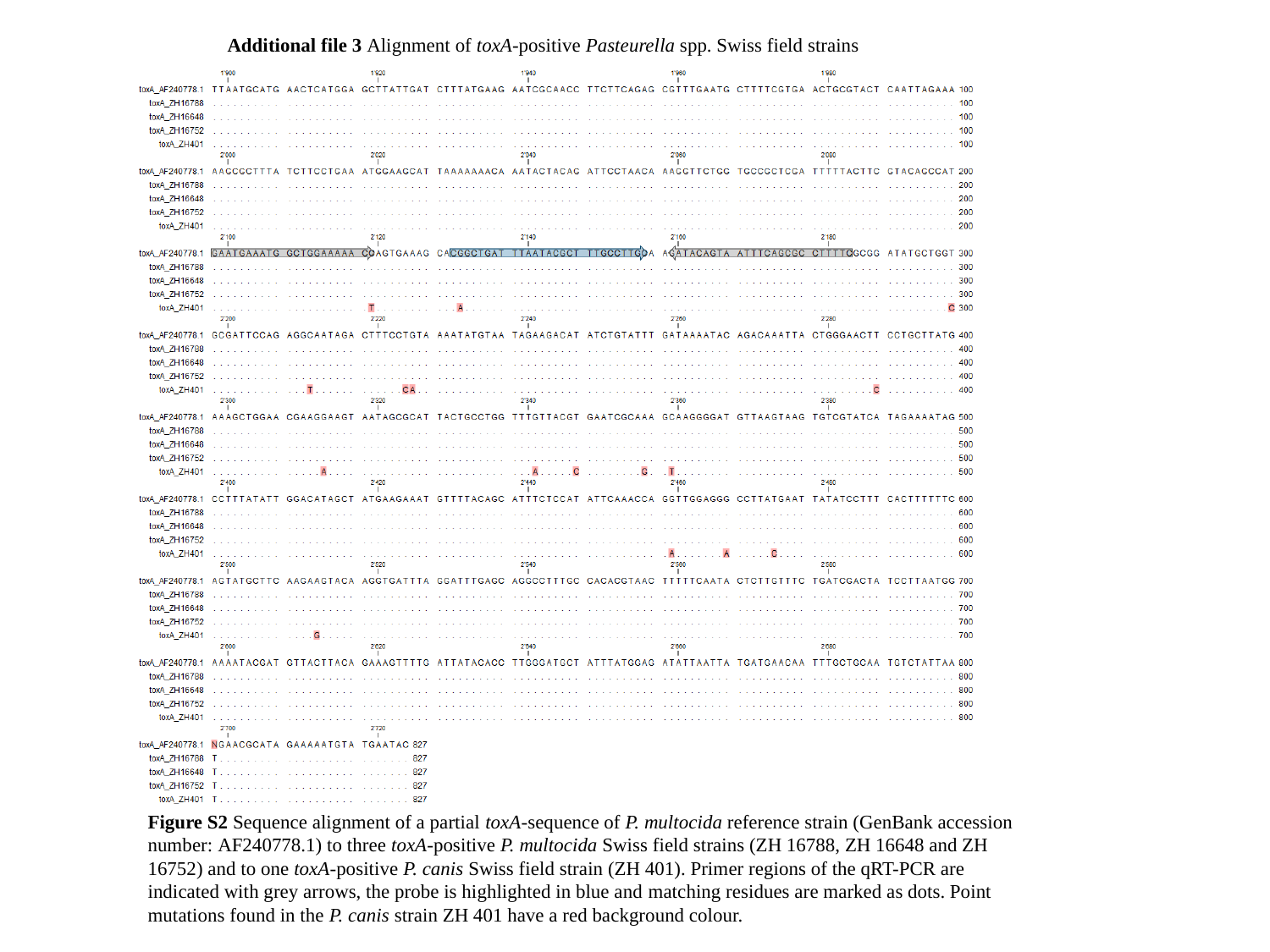

Additional file 3 Alignment of toxA-positive Pasteurella spp. Swiss field strains
Figure S2 Sequence alignment of a partial toxA-sequence of P. multocida reference strain (GenBank accession number: AF240778.1) to three toxA-positive P. multocida Swiss field strains (ZH 16788, ZH 16648 and ZH 16752) and to one toxA-positive P. canis Swiss field strain (ZH 401). Primer regions of the qRT-PCR are indicated with grey arrows, the probe is highlighted in blue and matching residues are marked as dots. Point mutations found in the P. canis strain ZH 401 have a red background colour.
